# Peripheral neuron phenotypes of familial dysautonomia are rescued by AAV-mediated gene therapy

**DOI:** 10.1007/s10286-026-01206-3

**Published:** 2026-04-09

**Authors:** Hsueh-Fu Wu, Tripti Saini, Jennifer Art, William Delaney, Frances Lefcort, Nadja Zeltner

**Affiliations:** 1 Center for Molecular Medicine, University of Georgia, Athens, GA USA; 2 Department of Biochemistry and Molecular Biology, University of Georgia, Athens, GA USA; 3 Biomedical and Translational Sciences Institute, Neuroscience Program, University of Georgia, Athens, GA 30602 USA; 4 Tikun Therapeutics and Familial Dysautonomia Foundation, New York, NY USA; 5 Department of Cellular Biology, University of Georgia, Athens, GA USA

Familial dysautonomia (FD) is a rare genetic, neurodevelopmental, and neurodegenerative disorder, where a homozygous mutation in the *ELP1* gene (IVS20+6T>C) is responsible for defects and symptoms found in 99% of patients [1]. FD primarily affects the peripheral nervous system (PNS) [2], including the autonomic and sensory nervous systems (ANS, SNS) [3]. The ANS regulates unconscious physiological responses and maintains body homeostasis, such as heart rate, blood pressure, gland secretion, and breathing, which are vital for bodily function. The SNS is the key mediator that processes and relays sensory information from the internal and external environment to the brain, including limb position, temperature, and pain.

In FD, defects of the ANS and SNS manifest in patients as symptoms such as dysregulation of heart rate and blood pressure, flushing, and abnormal sweating. Physical or emotional stress can further trigger life-threatening episodes called dysautonomic crises, characterized by hypertension, vomiting/retching, and behavioral changes [1]. Postganglionic sympathetic neurons (symNs) of the ANS, which control the fight-or-flight response and mediate autonomic functions, have been found to be decreased by about 30% in patients with FD [2]. FD was originally described as a disorder of afferent baroreflex failure [4]. Because sympathetic outflow is not inhibited, sympathetic activity is increased, explaining the hypertension. However, plasma noradrenaline levels are normal, unlike in other autonomic neuropathies, where they are typically reduced. Furthermore, the fact that non-FD patients with baroreflex failure mainly suffer from blood pressure instability, but do not experience other FD crisis symptoms (vomiting/retching and behavioral changes) suggests symN intrinsic defects in FD. Clinical symptoms were thought to largely relate to dopamine release, rather than noradrenaline, due to a bottleneck at the rate-limiting step of dopamine β-hydroxylase, which fails to be upregulated in FD [1]. In addition, we previously reported that symNs derived from patients with FD are spontaneously hyperactive [5]. This re-enforced clinical microneuropathy measurements in patients with FD [6]. Patients with FD also have difficulties sensing temperature/pain and suffer from gait ataxia, which is attributed to reduced sensory neuron numbers in patients’ sensory ganglia [2]. While patients with FD are born with symptoms, for example, difficulties swallowing, which leads to problems with feeding, research over the past decades has shown that PNS neurons also degenerate over time, leading to worsening of symptoms throughout life [1]. FD is also marked by the progressive death of retinal ganglion cells [7].

ELP1 belongs to the transcriptional elongator complex, which regulates tRNA modification; thus, it is critical for both transcription and translation [8]. The founder point mutation in the splice site of intron 20 of *ELP1* in FD results in a significant decrease of functional ELP1 RNA and protein [1]. Recent gene therapy approaches utilizing antisense oligonucleotides (ASOs) and small nuclear RNAs have shown promising results in correcting *ELP1* expression, which may rescue or mitigate FD disease phenotypes [9, 10]. Broadly, ASOs are short, single-stranded DNA/RNA analogues with the ability to bind to target RNA and modulate splicing signals on pre-mRNA [11]. In FD, they work by targeting the mutation on *ELP1* mRNA and correcting the defective splicing, potentially restoring cellular abnormalities [12]. Recently, Schultz et al. used adeno-associated virus (AAV) in the FD mouse model, where progressive death of retinal ganglion cells and axon degeneration occurs. This phenotype is similar to that of patients with FD who experience vision loss later in life. Schultz et al. sought to use gene replacement therapy of *ELP1* to treat the optic neuropathy in FD mice. AAVs were used to deliver a functional murine *Elp1* gene (AAV2-U1a-*Elp1*) by intravitreal injections [13]. As a result, they observed significantly improved retinal ganglion cell survival in FD mouse eyes compared to the untreated group. The enhanced green fluorescent protein (eGFP) control group (AAV2-U1a-eGFP) also showed significant protective effects albeit less than the murine Elp1-AAV2 group [13].

While this exciting work shows that AAV-mediated gene therapy can be effective in a mouse model, it is still unknown whether this is a viable therapeutic for human patients with FD. Human pluripotent stem cells (hPSCs), including embryonic stem cells (ESCs, derived from the blastocyst) and induced pluripotent stem cells (iPSCs, reprogrammed from adult somatic cells), have been prosperously applied to study human diseases in recent years [14]. hPSCs can self-renew in vitro, which provides an unlimited source of human, untransformed cells for research. By differentiating hPSCs, derived from a patient, into the desired cell types that are affected in the patient, scientists are able to model specific cellular and molecular defects contributing to the disease [14]. This technology, however, rests on the premise that reliable differentiation protocols to produce the desired cell types have been established. We have developed highly efficient, stable, and reproducible methods to differentiate multiple PNS lineages from hPSCs, including symNs, parasympathetic neurons, and sensory neurons [15–17]. We employed these tools to study FD disease mechanisms using healthy controls and hPSC models derived from patients with FD. We found that in FD, the progenitors of the PNS—neural crest cells—were significantly impaired, leading to reduced neuron numbers in the sympathetic and sensory lineage [15, 18]. We further described that FD symNs displayed spontaneous hyperactivity [15], which was rescued by some currently used FD treatments (dexmedetomidine) and novel compounds (clozapine-N-oxide). Using this model, we further described severe developmental and neurodegenerative defects in FD sensory neurons, which allowed us to conduct drug screening and drug discovery [18]. Our hPSC-based FD models, therefore, have been shown to be a critical tool to assess potential therapies for FD.

The promising mouse studies from Schultz et al. combined with our expertise led us to test the therapeutic effects of this gene replacement approach in our human FD PNS models. We first asked whether gene therapy can rescue FD symN hyperactivity [15]. We employed previously published hPSC-differentiation protocol to generate symNs [19]. Briefly, hPSCs undergo a 10-day neural crest cell induction, followed by a spheroid stage for neural crest purification and expansion. Finally, neural crest cells are replated and differentiated into postganglionic, functional symNs expressing tyrosine hydroxylase and peripherin. AAV carrying healthy human *ELP1* (AAV2-U1a-h*ELP1*) was added to FD hPSC-derived symNs on day 20 of differentiation, the stage when symN differentiation is complete, while the FD hyperactivity phenotype is not yet observed (it is detected after day 30) [15]. Experiments were performed on 96-well multielectrode array (MEA, by AxionBiosystems) plates with transparent bottoms (Fig. 1A), enabling us to monitor both the efficiency of viral transduction and neural activity at the same time. On day 20 of differentiation, we treated FD symNs with AAV2-U1a-h*ELP1* or AAV2-U1a-*eGFP* control, respectively, at a multiplicity of infection (MOI) of 400,000 (2.7 × 10^12^ vg/ml for AAV2-U1a-*ELP1* and 8.8 × 10^12^ vg/ml for AAV2-U1a-*eGFP*), in 30 µl symN culture medium/96 well. Six hours after the transduction, 120 µl fresh symN culture medium was added to each well. The next day (day 21), a complete medium change was performed to remove the AAV. On day 30, we compared the neural activity of untreated, AAV2-U1a-*ELP1*, or AAV2-U1a-*eGFP* treated FD symNs to healthy symNs and performed additional assays. On day 30, AAV2-U1a-*eGFP* treated FD symNs were successfully transduced, where about 60% of total cells were eGFP^+^ (Fig. 1A, B). All symN groups showed a similar level of cell density (Fig. 1C), which excludes the possibility that the difference in neural activity is caused by uneven cell numbers or possible viral toxicity. We next examined the level of *ELP1* mRNA splicing via RT-qPCR in each group. Compared to untreated and AAV2-U1a-*eGFP*-treated FD symNs, AAV2-U1a-*ELP1*-treated FD symNs showed a dramatic rescue effect in *ELP1* splicing, not significantly different from healthy symNs (Fig. 1D). Moreover, immunofluorescence staining shows an increase in ELP1 expression in AAV2-U1a-*ELP1*-treated FD symNs compared to untreated and AAV2-U1a-*eGFP*-treated FD symNs (Fig. 1E). Lastly, we analyzed neural activity among each group by MEA. Compared to healthy symNs, untreated FD symNs were hyperactive as previously reported [15] AV2-U1a-*ELP1* treatment significantly reduced this hyperactivity phenotype, to levels of the healthy control (Fig. 1F). Interestingly, similar to the results by Schultz et al., we also observed a mild level of rescue effect in AAV2-U1a-*eGFP* treated FD symNs, although not significantly different from untreated FD symNs (Fig. 1F). The similarity of the rescue effect on FD iPSC-derived symNs and FD mouse retina previously reported by Schultz et al. again highlights the utility and reliability of hPSC-based disease modeling on identifying tissue-specific pathologies and novel treatments in neurological disorders. Taken together, our data demonstrates that AAV2 gene therapy is well tolerated and rescues the ELP1 splicing deficiency and neuronal hyperactivity phenotype in human FD symNs, supporting its potential as a therapeutic for patients with FD (Fig. 1G).

**Fig. 1:**
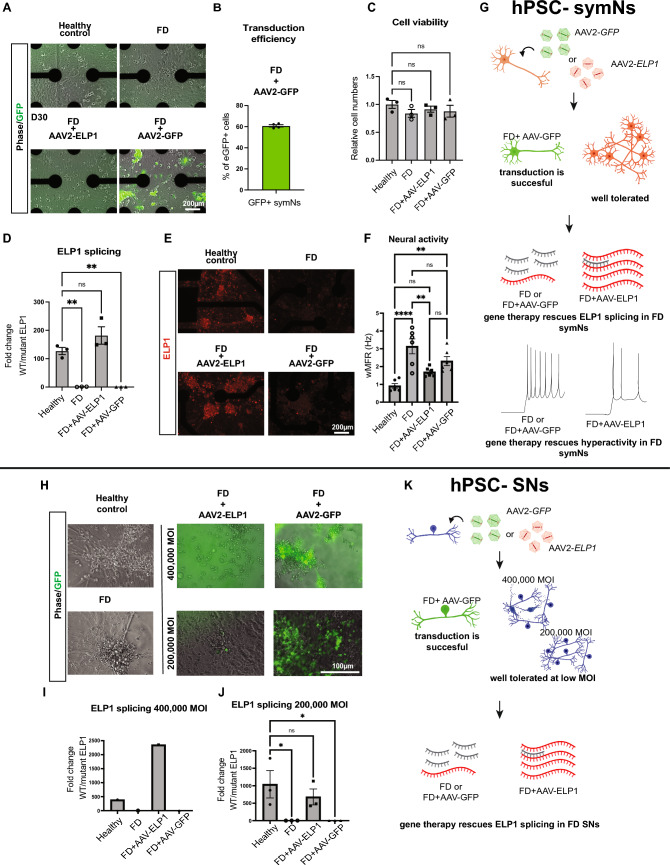
Examination of AAV-based gene therapy in human PNS neurons in FD. **A** Phase contrast and fluorescence images show symN morphology and eGFP expression/transduction of the neurons plated on MEA plates and assessed on day 30. Black strings/dots in the wells are the electrodes of the MEA plate. **B** The y-axis shows the percentage of eGFP^+^ cells of the total number of cells. **C** SymN cell numbers per well, relative to the non-transduced, healthy symNs. One-way ANOVA followed by Tukey’s multiple comparisons, where ns is non-significant, * is *p* ≤ 0.05, ** is *p* ≤ 0.01, *** is *p* ≤ 0.001, **** is *p* ≤ 0.0001. **D** RT-qPCR analysis for the ratio of wild type to mutant *ELP1* splicing in symNs. One-way ANOVA followed by Tukey’s multiple comparisons, *n* = 3 biological replicates, where ns is non-significant, * is *p* ≤ 0.05, ** is *p* ≤ 0.01, *** is *p* ≤ 0.001, **** is *p* ≤ 0.0001. **E** Fluorescence images show expression of wild-type ELP1 protein in symNs plated on MEA plates and fixed on d35. **F** MEA-based electrophysiological activity comparison. Data is presented as the weighted mean firing rate (wMFR, Mean firing rate multiplied by the number of active electrodes in a well). One-way ANOVA followed by Tukey’s multiple comparisons, *n* = 6 biological replicates, where ns is non-significant, * is *p* ≤ 0.05, ** is *p* ≤ 0.01, *** is *p* ≤ 0.001, **** is *p* ≤ 0.0001. **G** Summary graphic of AAV mediated gene therapy in FD symNs. **H** Phase contrast and fluorescence images showing the SN morphology and eGFP expression at 400,000 and 200,000 MOIs on a regular 96-well plate, on day 30 of SN differentiation. **I** RT-qPCR analysis for the ratio of wild type to mutant *ELP1* splicing in SNs at MOI 400,000. *n* = 1 biological replicate. **J** RT-qPCR analysis for the ratio of wild-type to mutant *ELP1* splicing in SNs at 200,000 MOIs. One-way ANOVA followed by Tukey’s multiple comparisons, *n* = 3 biological replicates, where ns is non-significant, * is *p* ≤ 0.05, ** is *p* ≤ 0.01, *** is *p* ≤ 0.001, **** is *p* ≤ 0.0001. **K** Summary graphic of AAV-mediated gene therapy in FD SNs

We next aimed to assess if these positive effects of AAV2-U1a-*ELP1* on FD are also present during sensory neuron (SN) development. SN differentiation was performed as previously described on regular 96-well plates [20]. Briefly, hPSCs undergo a 12-day neural crest induction. Neural crest cells are then dissociated and replated, followed by differentiation into SNs with dorsal root ganglion-like composition of mechanoreceptors, nociceptors, and proprioceptors. On day 14 of differentiation, at the early immature SN stage, the FD groups were transduced with AAV2-U1a-*ELP1* or AAV2-U1a-*eGFP* at the same MOI of 400,000 (2.7 × 10^12^ vg/ml for AAV2-U1a-*ELP1* and 8.8 × 10^12^ vg/ml for AAV2-U1a-eGFP). On day 30, we observed that the SNs were successfully transduced at this concentration, but the cells were stressed in both AAV2-U1a-*ELP1* or AAV2-U1a-eGFP control treatments (Fig. 1H, top). By performing RT-qPCR, we also observed that *ELP1* expression was very high in AAV2-U1a-*ELP1* (Fig. 1I). Thus, we next treated the FD neurons using an MOI of 200,000 (1.35 × 10^12^ vg/ml for AAV2-U1a-*ELP1* and 4.4 × 10^12^ vg/ml for AAV2-U1a-eGFP). On day 30, we confirmed effective viral transduction by observing eGFP expression in AAV2-U1a-*eGFP*-treated FD SNs and the cells were not stressed (Fig. 1H, bottom). RT-qPCR analysis confirmed *ELP1* splicing rescue in AAV2-U1a-*ELP1* transduced FD SNs (Fig. 1J). Our data suggest that AAV2 gene therapy is well tolerated and rescues FD *ELP1* deficiency in human FD SNs, further supporting that it can impact multiple PNS tissue types effected by FD (Fig. 1K).

While our study demonstrates a first step in utilizing hPSC-based disease modeling to show that AAV-mediated gene therapy may have restorative effects for FD peripheral phenotypes, it is not without limitations. In the current study, while there was no demonstrated cytotoxicity associated with the high MOI, SNs did appear stressed, which was resolved at a lower MOI. AAV transduction itself can be a stressor for cells, leading to non-specific effects, which should be further explored. Interestingly, we see a partial rescue of FD hyperactivity in symNs with the control AAV2-U1a-eGFP vector, which could be explained with the help of recent evidence that the AAV genome itself can affect hPSC-derived neuron function [21]. Exploration of this phenomenon will be warranted. Future work to further demonstrate the therapeutic potential of AAV-mediated gene therapy will include extended study of phenotype rescue beyond 10 days after initial AAV transduction, as well as introducing gene therapy after the onset of symN hyperactivity. Additionally, we would like to expand the scope of our assays, particularly in SNs, to more deeply characterize the extent to which gene therapy can rescue functional disease phenotypes including morphology, long-term survival, and degeneration.

Together, our data show that hPSC-based disease modeling can be used as a reliable human platform to assess potential rescue effects of AAV-based gene therapy approaches, a first step in determining its promise for patients with FD. We demonstrate the promising therapeutic effect of AAV2-based gene therapy, promoting splicing rescue in peripheral neurons and normalizing function in FD symNs, supporting it as a potential treatment for FD.

## Data Availability

All data generated or analyzed in this study are included in this published article. Raw data points will be deposited in the figshare.com database.
